# Supplementary material for: Dynamic metabolic modeling of Streptomyces clavuligerus in complex medium highlights nutrient-dependent metabolic transitions associated with clavulanic acid biosynthesis
Source: PLoS One. 2026 Feb 2;21(2):e0342057. doi: 10.1371/journal.pone.0342057 (PMC12863558; doi:10.1371/journal.pone.0342057)
Supplement: S1 File — (PDF) [file pone.0342057.s001.pdf]

Supporting information S1: *Streptomyces clavuligerus* unstructured kinetic model construction

Table of contents

1. Unstructured kinetic model construction ……………………………………………1

1.1. Cellular growth model…………………………………………………………...1

1.2. Substrate consumption model……………………………………………………2

1.3. Clavulanic acid production model………………………………………………2

1.4. Experimental rates calculation…………………………………………………...3

2. Model parameter estimation……………………………………………………....3

3. Results…………………………………………………………………………......4

4. References…………………………………………………………………….......6

1. Unstructured kinetic model construction

1.1. Cellular growth model

A typical fermentation medium used in CA production encompasses a variety of carbon and nitrogen substrates, notably amino acids derived from free organic nitrogen sources, ammonia, and glycerol. The uptake of these substrates may occur sequentially or concurrently. In this study, GLYCAS-5 medium comprising glycerol, free amino acids, phosphate and salts was used. Consequently, it is imperative for process models to accurately forecast the growth, CA synthesis, and sequential uptake patterns of amino acids [1,2].

Accordingly, we postulated that the organism has access to five distinct categories of substrates: (i) the main carbon source as glycerol, Glyc, (ii) seven amino acids supporting growth during the exponential phase (0-40 h), AA_i, (iii) nine amino acids consumed at the latter stage of the exponential phase (Glu-, Asp-, Ser-, Phe-, Trp-, Ala- and His-L) and thereafter, $AA_{,j}$,(Pro-, Thr-, Lys-, Cys-, Arg-, Leu-,Tyr-, Gly- and Val-L) (iv) organic nitrogen sources such as ammonia, NH_4, and (v) phosphate, PO_4. The fractional rate of growth associated with glycerol, μ_{Glyc}, and amino acids, $\mu_{AA,i}$and $\mu_{AA,j}$, were modeled using modified Monod type kinetics as shown in equations 1 to 3. The total growth, μ, was expressed as the sum of growth rates associated with glycerol as the main carbon source and the contribution of amino acids (Eq. 4). These constitutive equations are shown as follows:

$$\mu_{Glyc} = \mu_{max,Glyc} \frac{Glyc}{K_{Glyc} \cdot X + Glyc} \tag{1}$$

$$\mu_{AA,j} = \mu_{max,AA,j} \frac{{AA_j}^2}{K_{AA,j} + AA_j} \tag{2}$$

$$\mu_{AA,i} = \mu_{max,AA,i} \frac{{AA_i}^2}{K_{AA,i} + AA_i} \tag{3}$$

$$\mu = \mu_{Glyc} + \sum_{i=1}^{7} \mu_{AA,i} + \sum_{j=1}^{9} \mu_{AA,j} \tag{4}$$

$$\frac{dX}{dt} = \mu \cdot X - k_{d,X} \cdot X \cdot fc_1 \tag{5}$$

1.2.Substrate consumption model

Utilizing a simplified stoichiometric framework for biomass synthesis and employing non-steady-state mass balances, an unstructured model was proposed to describe the biomass (X) synthesis, nutrient consumption (glycerol, Glyc, amino acids, AA_i and AA_j, ammonia, NH_4, and phosphate, PO_4) and by-product excretion (CA) [3]. The resulting set of ordinary differential equations (ODE) describing the mass balances is presented in equations 5 to 11.

$$\frac{dGlyc}{dt} = -\frac{1}{Y_{X/Glyc}} \mu_{Glyc} \cdot X \tag{6}$$

$$\frac{dAA_i}{dt} = -\mu_{AA,i} \cdot X \tag{7}$$

$$\frac{dAA_j}{dt} = -\mu_{AA,j} \cdot X \cdot fc_1 \tag{8}$$

$$\frac{dNH_4}{dt} = -\frac{1}{Y_{X/NH_4,Glyc}} \mu_{Glyc} \cdot X \tag{9}$$

$$\frac{dPO_4}{dt} = -\frac{1}{Y_{X/PO_4,Glyc}} \mu_{Glyc} \cdot X \tag{10}$$

For modeling, it was assumed that the microorganism grew on glycerol according to the Monod model and that cell death began when glycerol was nearly depleted (below a critical concentration C_{S1}<4.86). Regarding production, it was assumed that catabolic repression occurred during part of the growth phase (C_S> 23.6).

1.3. Clavulanic acid production model

Clavulanic acid formation began when the glycerol concentration reached a critical value C_{S2}, which is higher than C_{S1}. The function fc_1 is a "step function" that is initially zero and takes a value of 1 when the concentration falls below the critical level C_{S1}, setting up the onset of cell death. Similarly, fc_2 is a "step function" that is initially zero during catabolic repression and takes a value of 1 when the glycerol concentration drops below the critical value C_{S2}. Cellular death and product degradation were considered to follow first-order kinetics regarding cellular (X) and product (CA) concentrations. These were defined according to the usual Monod rate expression where $k_{d,X}$ and k_P are the death rate constant and product degradation rate constant, respectively (Eqs. 5 and 11). The parameters of the kinetic model were identified by minimizing the least square error using the Levenberg-Marquardt method with experimental data of *S. clavuligerus* cultivations [1,3].

$$\frac{dCA}{dt} = \left(\frac{\mu}{Y_{X/CA}} + \beta\right) \cdot X \cdot fc_1 - k_P \cdot CA \cdot fc_2 \tag{11}$$

1.4. Experimental rates calculation

The extracellular component rates were calculated as follows and were subsequently used as constraints for the dynamic flux balance analysis.

$$q_{glyc} = \frac{dGlyc(t)}{dt}/X(t); \quad q_{PO4} = \frac{dC_{PO4}(t)}{dt}/X(t);$$
$$q_{NH4} = \frac{dC_{NH4}(t)}{dt}/X(t); \quad q_{CA} = \frac{dC_{CA}(t)}{dt}/X(t);$$
$$q_{AA,i} = \frac{dC_{AA,i}(t)}{dt}/X(t); \quad \mu = \frac{dX(t)}{dt}/X(t)$$

2. Model Parameter Estimation

A non-linear least squares method was employed to estimate the kinetic parameters of the models described in Equations (1), (4)–(7) using the minimize function from the SciPy library [4]. The models were simultaneously solved using the *odeint* function from SciPy to solve ordinary differential equations (ODEs) with the explicit Runge-Kutta method, along with an analysis of residual dispersion [5].

Mathematical expressions for the sum of squares for regression (SSR), total sum of squares

(SST) coefficient of determination (R^2) (Equation (8)) and sum of Squares for Error (SSE) were used to assess the deviation of experimental data obtained compared to simulated data. The kinetic parameter values obtained from experimental results were analyzed through Duncan's multiple comparisons with a confidence level of 95%, aiming to determine whether there were significant differences among the values obtained from different substrates.

The equations are as follows:

$$SSR = \sum_{i=1}^{n}(\hat{y_i} - \bar{y})^2 \ ; \ SST = \sum_{i=1}^{n}(y_i - \bar{y})^2$$

$$R^2 = \frac{SSR}{SST} = 1 - \frac{SSE}{SST} \ ; \ SSE = \sum_{i=1}^{n}(y_i - \hat{y_i})^2$$

Where $\hat{y_i}$ represents each simulated (or fitted) value from the model, $\bar{y}$ is the mean of the observed or predicted data y.

3. Results

The results of the parameter estimation for each variable in the unstructured kinetic model are summarized in Table S1, including statistical indicators of model performance such as the total sum of squares (SST), the sum of squared regression (SSR), and the coefficient of determination (R^2). In parallel, Fig. S1 displays the experimental and simulated time-course profiles for biomass, substrate consumption (glycerol, ammonia, phosphate), clavulanic acid production, and amino acid uptake, highlighting the model's predictive capability for each variable.

Table S1. Statistical summary of model fitting for each metabolite and biomass variable.

**Metabolite**	**SST**	**SSR**	**R^2**	**Metabolite**	**SST**	**SSR**	**R^2**
Biomass	226.5372	11.7011	0.94835	Cysteine	0.7202	0.0037	0.9948
Glycerol	40791.0974	554.4361	0.98641	Leucine	2.6636	0.0099	0.9963
Phosphate	224.9822	129.8286	0.42294	Lysine	1.1368	0.3210	0.7176
Ammonia	9903.9176	2283.7981	0.76940	Proline	3.2585	0.0057	0.9983

Clavulanic acid	0.0361	0.0005	0.98531	Histidine	0.0250	0.0036	0.8540
Glutamate	7.2413	0.0044	0.99939	Tyrosine	0.1402	0.0002	0.9989
Aspartate	2.1572	0.0000	0.99999	Glycine	0.4917	0.0003	0.9993
Serine	0.9669	0.0000	0.99999	Valine	0.1407	0.0000	0.9998
Alanine	0.7479	0.0014	0.99815	Tryptophan	0.0000	0.0000	0.8185
Phenylalanine	0.2801	0.0113	0.95963	Histidine	0.0250	0.0036	0.8540
Arginine	0.2311	0.0429	0.81437				

SSR: sum squared regression, SST: Total sum squares, R^2: Correlation coefficient

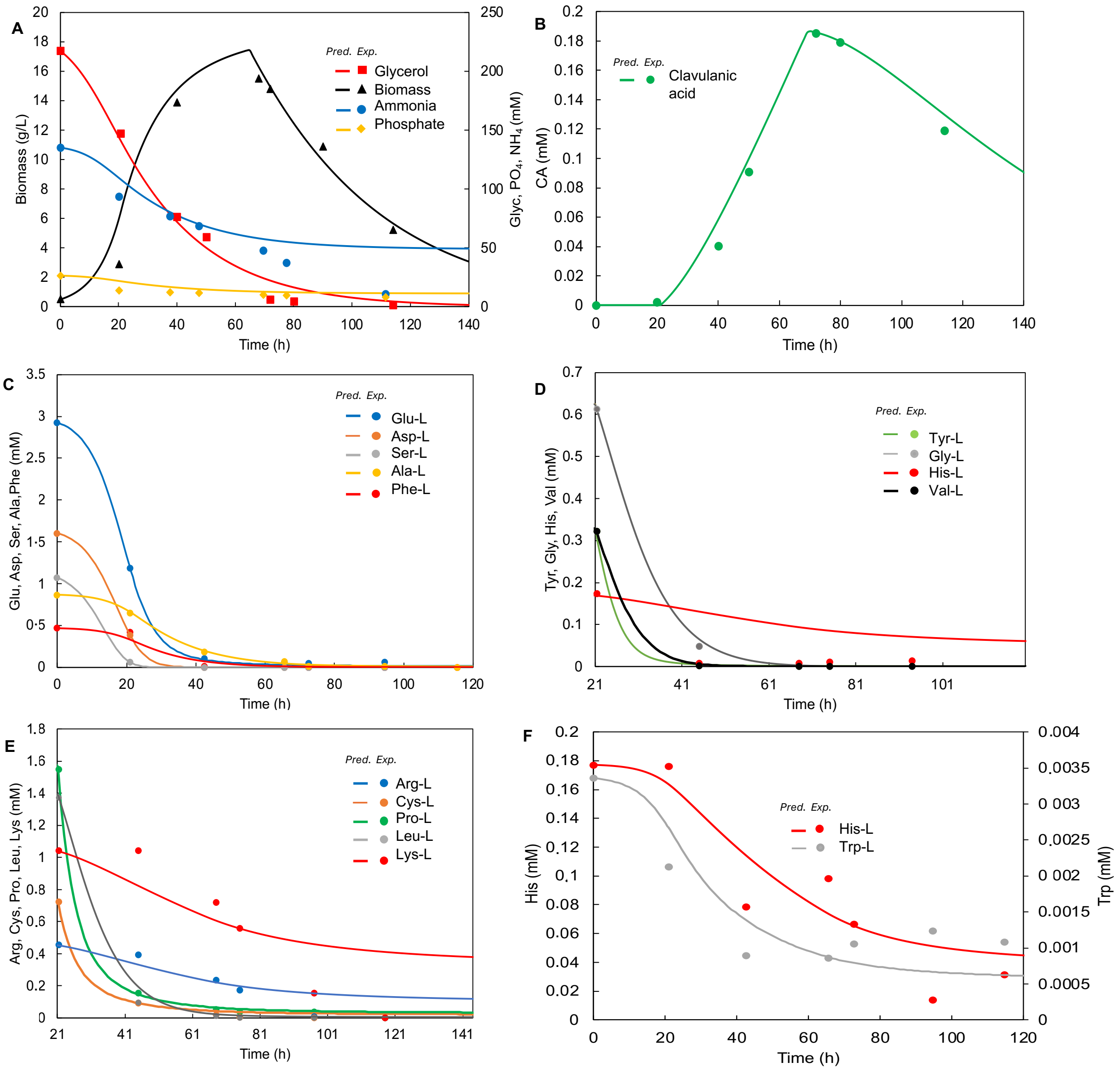

Figure S1. Experimental and predicted time-course data for (**A**) biomass formation and substrate (glycerol, ammonia, phosphate) consumption, (**B**) clavulanic acid production, and (**C–F**) amino acid uptake by *Streptomyces clavuligerus*. Experimental data points are shown as markers; model predictions are shown as continuous lines.

References

1. Bapat PM, Bhartiya S, Venkatesh KV, Wangikar PP. Structured kinetic model to represent the utilization of multiple substrates in complex media during rifamycin B fermentation. Biotechnol Bioeng. 2006;93: 779–790. doi:10.1002/bit.20767

2. Bapat PM, Sohoni SV, Moses TA, Wangikar PP. A cybernetic model to predict the effect of freely available nitrogen substrate on rifamycin B production in complex media. Appl Microbiol Biotechnol. 2006;72: 662–670. doi:10.1007/s00253-006-0341-6

3. Baptista-Neto A, Gouveia ER, Badino-Jr AC, Hokka CO. Phenomenological model of the clavulanic acid production process utilizing *Streptomyces clavuligerus*. Braz J Chem Eng. 2000;17: 809–818. doi:https://doi.org/10.1590/S0104-66322000000400043

4. Virtanen P, Gommers R, Oliphant TE, Haberland M, Reddy T, Cournapeau D, et al. SciPy 1.0: fundamental algorithms for scientific computing in Python. Nat Methods. 2020;17: 261–272. doi:10.1038/s41592-019-0686-2

5. Feng X, Xu Y, Chen Y, Tang YJ. Integrating Flux balance analysis into kinetic models to decipher the dynamic metabolism of *Shewanella oneidensis* MR-1. Reed JL, editor. PLoS Comput Biol. 2012;8: e1002376. doi:10.1371/journal.pcbi.1002376
