# Supplementary material for: Dynamic metabolic modeling of Streptomyces clavuligerus in complex medium highlights nutrient-dependent metabolic transitions associated with clavulanic acid biosynthesis
Source: PLoS One. 2026 Feb 2;21(2):e0342057. doi: 10.1371/journal.pone.0342057 (PMC12863558; doi:10.1371/journal.pone.0342057)
Supplement: S3 File — (PDF) [file pone.0342057.s003.pdf]

Supporting information S3: Correlation between the kinetic model and dynamic flux balance analysis (dFBA) simulations using the genome-scale metabolic model iLT1021 of *Streptomyces clavuligerus*

This supplementary material presents the correlation analysis between experimentally derived kinetic model outputs and predicted exchange fluxes obtained through dynamic flux balance analysis (dFBA) simulations, using the refined iLT1021 genome-scale metabolic model. The analysis includes comparisons for growth rate, glycerol, ammonia, and phosphate uptake rates.

S1 Fig. Correlation between experimentally derived kinetic model outputs and predicted exchange fluxes obtained from dynamic flux balance analysis (dFBA) simulations, using the refined genome-scale metabolic model iLT1021 of *Streptomyces clavuligerus*. The plots show the comparison of predicted versus observed values for (**A**) specific growth rate, (**B**) glycerol uptake rate, (**C**) ammonia uptake rate, (**D**) phosphate uptake rate, (**E**) Clavulanic acid excretion rate, **(F-U**) Amino acid uptake rates. Coefficients of determination (R^2) indicate the level of agreement between both modeling approaches.

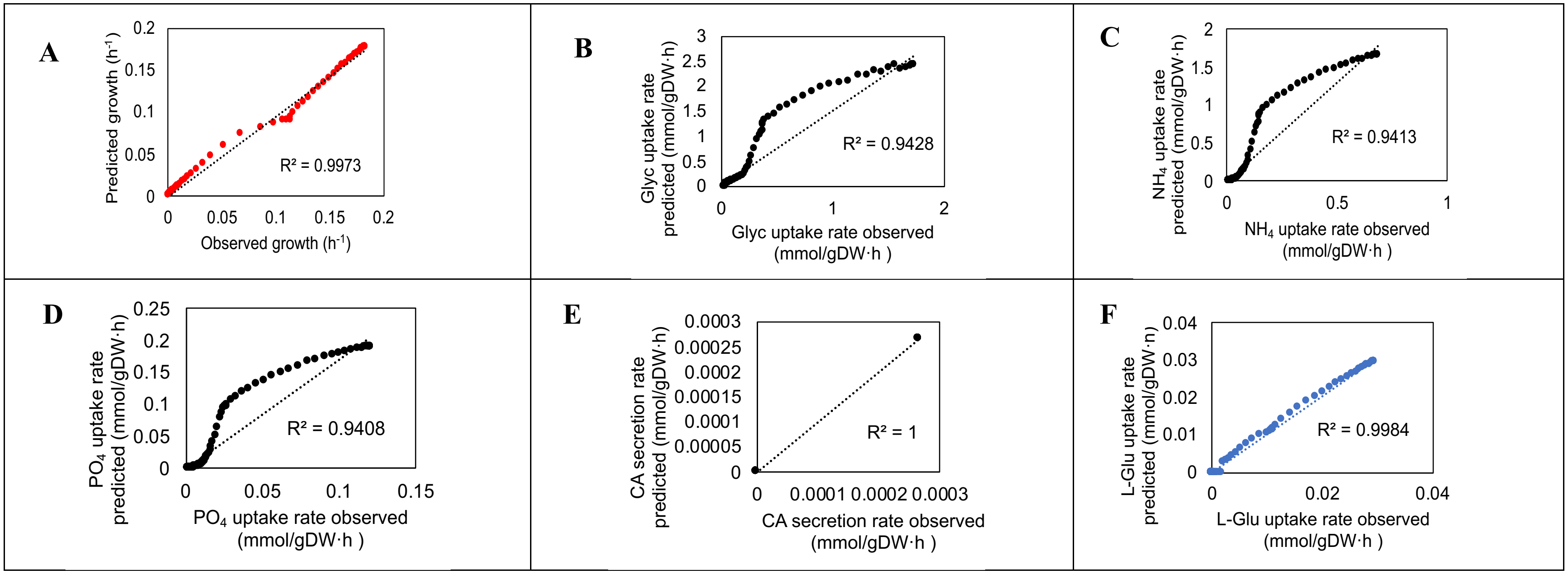

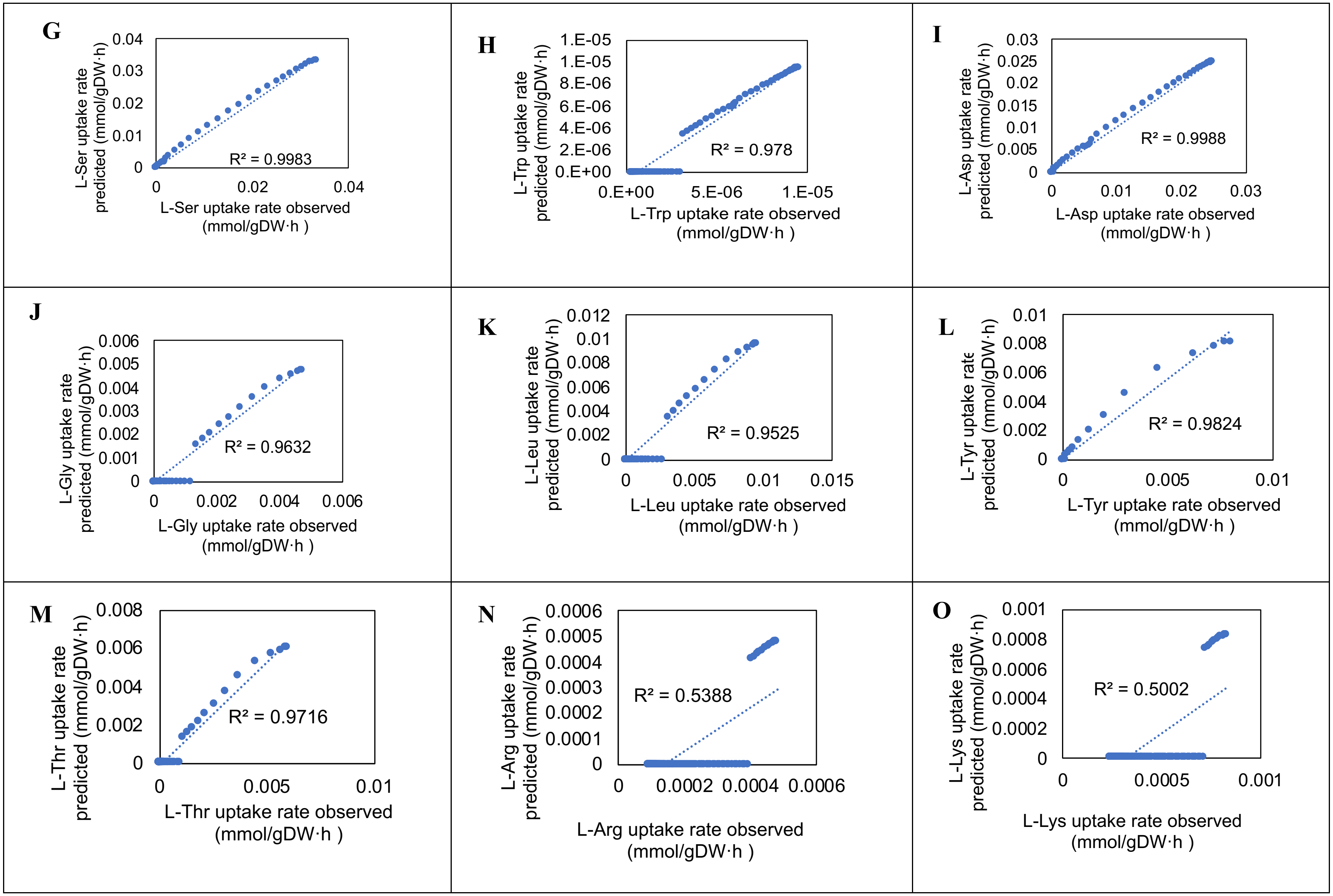
G
L-Ser uptake rate predicted (mmol/gDW·h)
0.04
0.03
0.02
0.01
0
R² = 0.9983
0
0.02
0.04
L-Ser uptake rate observed (mmol/gDW·h)
H
L-Trp uptake rate predicted (mmol/gDW·h)
1.E-05
1.E-05
8.E-06
6.E-06
4.E-06
2.E-06
0.E+00
R² = 0.978
0.E+00
5.E-06
1.E-05
L-Trp uptake rate observed (mmol/gDW·h)
I
L-Asp uptake rate predicted (mmol/gDW·h)
0.03
0.025
0.02
0.015
0.01
0.005
0
R² = 0.9988
0
0.01
0.02
0.03
L-Asp uptake rate observed (mmol/gDW·h)
J
L-Gly uptake rate predicted (mmol/gDW·h)
0.006
0.005
0.004
0.003
0.002
0.001
0
R² = 0.9632
0
0.002
0.004
0.006
L-Gly uptake rate observed (mmol/gDW·h)
K
L-Leu uptake rate predicted (mmol/gDW·h)
0.012
0.01
0.008
0.006
0.004
0.002
0
R² = 0.9525
0
0.005
0.01
0.015
L-Leu uptake rate observed (mmol/gDW·h)
L
L-Tyr uptake rate predicted (mmol/gDW·h)
0.01
0.008
0.006
0.004
0.002
0
R² = 0.9824
0
0.005
0.01
L-Tyr uptake rate observed (mmol/gDW·h)
M
L-Thr uptake rate predicted (mmol/gDW·h)
0.008
0.006
0.004
0.002
0
R² = 0.9716
0
0.005
0.01
L-Thr uptake rate observed (mmol/gDW·h)
N
L-Arg uptake rate predicted (mmol/gDW·h)
0.0006
0.0005
0.0004
0.0003
0.0002
0.0001
0
R² = 0.5388
0
0.0002
0.0004
0.0006
L-Arg uptake rate observed (mmol/gDW·h)
O
L-Lys uptake rate predicted (mmol/gDW·h)
0.001
0.0008
0.0006
0.0004
0.0002
0
R² = 0.5002
0
0.0005
0.001
L-Lys uptake rate observed (mmol/gDW·h)

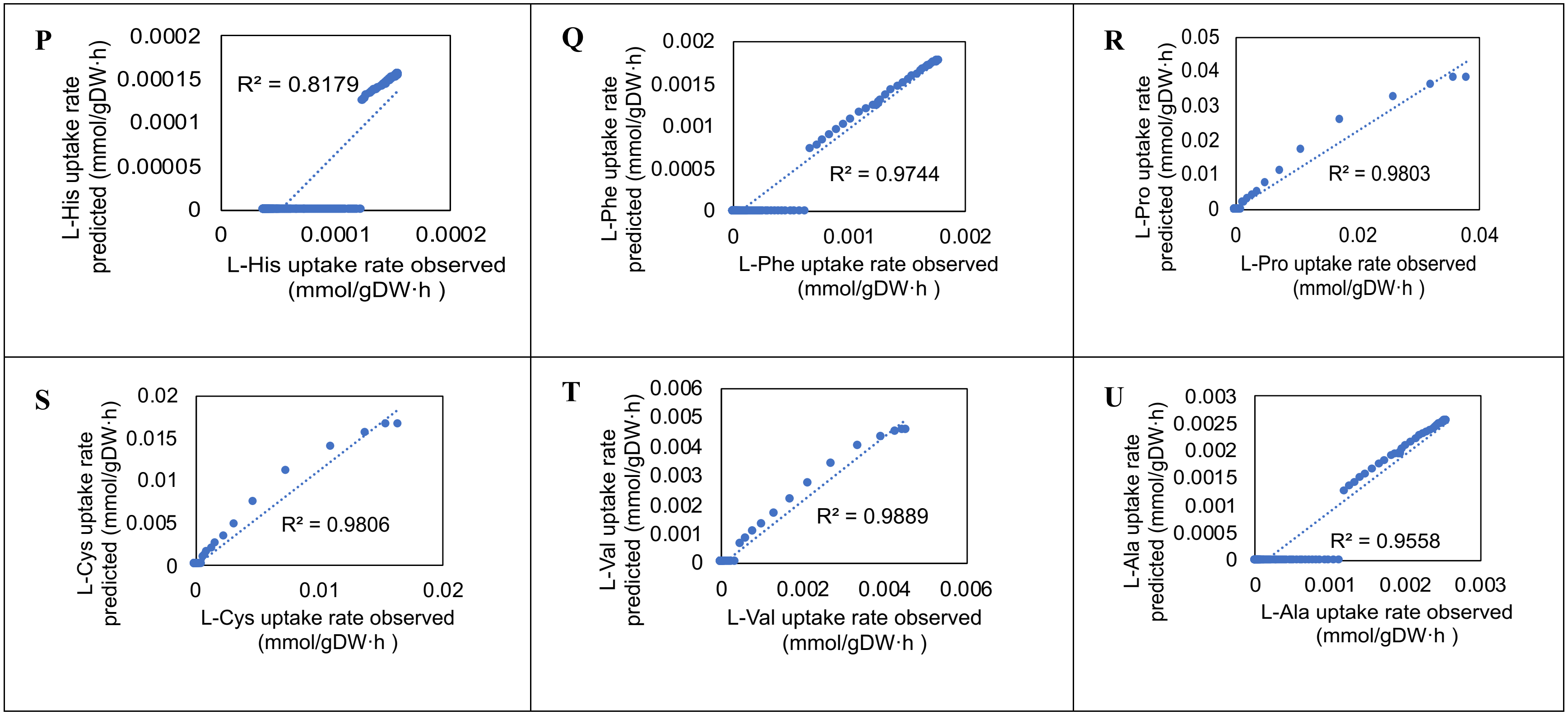
P
L-His uptake rate predicted (mmol/gDW·h)
0.0002
0.00015
0.0001
0.00005
0
R² = 0.8179
0
0.0001
0.0002
L-His uptake rate observed (mmol/gDW·h)
Q
L-Phe uptake rate predicted (mmol/gDW·h)
0.002
0.0015
0.001
0.0005
0
R² = 0.9744
0
0.001
0.002
L-Phe uptake rate observed (mmol/gDW·h)
R
L-Pro uptake rate predicted (mmol/gDW·h)
0.05
0.04
0.03
0.02
0.01
0
R² = 0.9803
0
0.02
0.04
L-Pro uptake rate observed (mmol/gDW·h)
S
L-Cys uptake rate predicted (mmol/gDW·h)
0.02
0.015
0.01
0.005
0
R² = 0.9806
0
0.01
0.02
L-Cys uptake rate observed (mmol/gDW·h)
T
L-Val uptake rate predicted (mmol/gDW·h)
0.006
0.005
0.004
0.003
0.002
0.001
0
R² = 0.9889
0
0.002
0.004
0.006
L-Val uptake rate observed (mmol/gDW·h)
U
L-Ala uptake rate predicted (mmol/gDW·h)
0.003
0.0025
0.002
0.0015
0.001
0.0005
0
R² = 0.9558
0
0.001
0.002
0.003
L-Ala uptake rate observed (mmol/gDW·h)
