# Supplementary material for: Dynamic metabolic modeling of Streptomyces clavuligerus in complex medium highlights nutrient-dependent metabolic transitions associated with clavulanic acid biosynthesis
Source: PLoS One. 2026 Feb 2;21(2):e0342057. doi: 10.1371/journal.pone.0342057 (PMC12863558; doi:10.1371/journal.pone.0342057)
Supplement: Attachment — Submitted filename: Response_to_reviewers_Toro_et_al_2025_PlosOne.pdf [file pone.0342057.s006.pdf]

Response to reviewers

Manuscript ID: PONE-D-25-57065

Title: Dynamic metabolic modeling of *Streptomyces clavuligerus* in complex medium highlights nutrient-dependent metabolic transitions associated with clavulanic acid biosynthesis

Dear Academic Editor and Reviewers,

We greatly appreciate the positive assessment of the scientific soundness of the study and the constructive suggestions provided. All comments have been carefully addressed, resulting in substantial improvements in methodological clarity, figure presentation, and separation of experimental observations from interpretation. Below, we provide a detailed, point-by-point response. All changes have been incorporated into the revised manuscript and highlighted in the tracked-changes version.

Data Availability Statement

The Data Availability Statement has been updated to explicitly indicate that the amino acid interaction network reconstructed from the iLT1021 genome-scale metabolic model is publicly available in the Network Data Exchange (NDEx) repository under accession ID 4b9fe51c-e7f1-11f0-a218-005056ae3c32, in full compliance with PLOS ONE data availability requirements. All other relevant data are provided within the manuscript and Supporting Information files.

We believe that all reviewer comments have been fully addressed and that the revised manuscript meets the standards and scope of PLOS ONE. We appreciate the time and effort invested by the editor and reviewers and hope that the revised version will be suitable for publication.

Sincerely,

Leon Felipe

On behalf of all authors

Response to Reviewer #1

Methods Section

Reviewer comment:

1. Kinetic Modeling Description (Lines 137–144)

The description of the unstructured kinetic model is too brief for readers unfamiliar with such models. Please include a short explanation of:

- *how many differential equations were used,*
- *what each equation represents (biomass, glycerol, amino acids, CA, death),*
- *and the general parameter-fitting approach used.*

Relying solely on S1 File makes the Methods section difficult to follow.

Response:

We appreciate this observation. We have expanded the description of the unstructured kinetic model to improve clarity for readers who may not be familiar with this type of modeling. The revised Methods section now specifies: (i) the total number of differential equations included in the model, (ii) the role of each equation in describing biomass formation, substrate consumption, product formation, and cell decay, and (iii) the general parameter-fitting strategy based on nonlinear least-squares optimization. This information has been added directly to the Methods section (Lines 137–163), while the S1 File remains as a complementary resource containing the full mathematical formulation.

Reviewer comment:

2. Topological Analysis Workflow (Lines 146–161)

The workflow for constructing the amino-acid network from the iLT1021 GEM is not sufficiently explained. Please clarify:

- *how nodes and edges were defined,*
- *whether cofactors were excluded,*
- *how sequential reactions were collapsed into interactions.*

This added detail would help non-expert readers understand how the network was built.

Response:

We thank the reviewer for pointing out the need for additional methodological detail. We have expanded this section to clarify how the network was constructed from the iLT1021 GEM. The revised text now explains: (i) how nodes and edges were defined based on amino acid participation in metabolic reactions, (ii) that common cofactors (ATP, NAD(H), NADP(H), FAD, water, protons, etc.) were excluded to avoid spurious connections, and (iii) how consecutive reactions were collapsed into direct amino-acid interactions when they formed linear biosynthetic or degradation sequences. These details have been added to the Methods section (Lines 166–181).

Reviewer comment:

3. Robustness Analysis and CA Production (Lines 163–178)

It is unclear how CA production rates were obtained during Robustness Analysis. Since maximizing growth typically gives zero CA flux, I was unsure whether:

- *a second optimization step was applied,*
- *CA fluxes were constrained, or*
- *the values were directly taken from the growth simulation.*

Response:

We appreciate this important clarification request. In the revised manuscript, we now explicitly describe how CA fluxes were obtained during the Robustness Analysis. At each point of the RA, after maximizing biomass, we performed a secondary optimization step (maximizing CA production) while constraining the growth rate to the optimal value previously obtained. This two-step procedure allowed us to quantify the maximum CA flux compatible with the growth optimum. We did not impose any forced CA flux constraints during the first optimization step. These details have been added to the Methods section (Lines 204–219).

Reviewer comment:

4. High Biomass-Yield Coefficients (Table 1)

*Some biomass-yield coefficients (e.g., **YX/Glu = 32.7**, **YX/Ala = 14.4**, **YX/Cys = 15.9 gDW/mmol**) are much higher than typical microbial yields. It is unclear whether:*

- *the units are correct (gDW/mmol vs. gDW/mol),*
- *the values reflect empirical fitting rather than physiological yields,*

- *or scaling/normalization effects are involved.*

A short explanation in the Results or Discussion would help readers interpret these values correctly.

Response:

We appreciate the reviewer's observation. The biomass-yield coefficients reported in Table 1 are expressed in gDW per mmol, consistently with the formulation of the unstructured dynamic model and the mmol-based mass balances used for all substrates. These coefficients are not intended to represent physiological biomass yields in the classical sense. Instead, they act as effective fitted parameters that scale the contribution of each amino acid to biomass formation within a simplified multi-substrate kinetic framework.

In the GLYCAS-5 medium, amino acids differ markedly in concentration, uptake rates, and metabolic roles. Several of them function primarily as nitrogen donors or anabolic precursors rather than as main carbon or energy sources. As a consequence, relatively small consumptions expressed on a mmol basis can be associated with substantial biomass increases, leading to high numerical values of YX/i. Therefore, these coefficients reflect the relative contribution and timing of substrate utilization required to reproduce the experimental biomass dynamics, rather than true stoichiometric or physiological yields.

To avoid misinterpretation, we have added a clarification in the Results section explicitly stating the empirical and model-dependent nature of these parameters.

Discussion Section

Reviewer comment:

The Discussion is thorough and integrates multiple analytical approaches. However, a few areas require clarification.

5. Mixing Experimental Findings with Interpretation (Lines 464–472, 473–476, 477–487, 489–497, 508–516, 517–525)

In several places, measured experimental data (e.g., Fig. 4 depletion trends) are presented together with physiological interpretations or model predictions. For example, Lines 464–472 combine measured amino-acid depletion with inferred causes such as anabolic demand or peptide hydrolysis.

A similar mixing appears in discussions of:

- *nutrient triggers for stationary phase (Lines 473–476),*
- *hub-metabolite roles from topology (Lines 477–487),*
- *RA nitrogen-source performance (Lines 489–497),*
- *dFBA substrate and flux trends (Lines 508–525).*

Suggestion: *Please clearly separate observational results from interpretation, and explicitly indicate when statements derive from experimental data versus model predictions or literature reasoning.*

Reviewer response:

We thank the reviewer for this important and constructive comment. In response, we have thoroughly revised the manuscript to clearly separate observational experimental results from physiological interpretation, model-based predictions, and literature-supported reasoning across all the sections indicated.

Specifically, in Lines 464–472, all interpretative statements (e.g., preferential carbon/nitrogen utilization, anabolic demand, or peptide hydrolysis) have been removed from the Results section. This section now reports exclusively experimentally measured amino acid concentration profiles and depletion trends (Fig. 4). Corresponding physiological interpretations, model-based predictions, and literature-supported explanations (including references [9,14]) have been relocated to and are explicitly identified in the Discussion section.

A similar revision strategy has been consistently applied to the other sections highlighted by the reviewer. Experimental observations related to nutrient triggers for stationary phase (Lines 473–476), network topology and hub-metabolite analysis (Lines 477–487), robustness analysis of nitrogen-source performance (Lines 489–497), and dFBA-derived substrate and flux trends (Lines 508–525) are now presented independently in the Results section, with explicit reference to measured data, figures, or simulation outputs.

Physiological interpretations, model-derived insights, and broader biological implications are now clearly introduced in separate sentences or paragraphs and explicitly framed as interpretative reasoning supported by either model predictions or literature evidence [19,35,44–47]. In cases where unexpected experimental trends were observed (e.g., transient amino acid accumulation or lower-than-expected nitrogen-source performance), these are first described as observations, followed by clearly labeled interpretative explanations.

Overall, these revisions ensure a clear and consistent distinction between experimental observations, model-based predictions, and literature-supported interpretations throughout the manuscript, thereby improving both readability and scientific clarity.

Reviewer comment:

6. Missing Discussion of Unexpected Findings (Lines 473–487; 489–497; 273–279)

Some noteworthy discrepancies are not addressed, such as:

- *transient increases in certain amino acids early in growth,*
- *Lys/His showing poor CA support experimentally despite moderate predicted fluxes,*
- *low model fit for phosphate ($R^2 = 0.423$).*

A brief explanation or acknowledgement would help contextualize these deviations.

Reviewer response:

We thank the reviewer for pointing out these unexpected findings. We have revised the manuscript to explicitly acknowledge and contextualize the indicated discrepancies.

First, the transient increases observed for several amino acids during early growth (Lines 473–487) are now clearly described as experimental observations, followed by a brief literature-based discussion linking these patterns to peptide hydrolysis and transient intracellular amino acid release.

Second, the unexpectedly low effectiveness of lysine and histidine in supporting clavulanic acid production (Lines 489–497), despite moderate predicted fluxes in the robustness analysis, is now explicitly acknowledged. We discuss plausible explanations, including regulatory constraints or slower assimilation kinetics not fully captured by the model.

Finally, the relatively low model fit for phosphate (and ammonium) (Lines 273–279) is now addressed in the Discussion. We clarify that ammonium dynamics are influenced by intracellular amino acid deamination not represented in the unstructured kinetic model, while phosphate exerts complex regulatory control that is difficult to capture within a simplified modeling framework.

Together, these revisions provide appropriate context for the observed deviations while preserving the overall validity of the experimental results and modeling framework.

Reviewer comment:

7. Interpretation of High Yield Values (Lines 282–297)

Although high Yx values are discussed, the manuscript does not address:

- *why these values exceed typical physiological limits,*
- *whether they result from model-fitting constraints,*
- *or whether they influence model behavior.*

Please add a concise clarification.

Reviewer response:

We thank the reviewer for pointing out the need to clarify the interpretation of the high biomass-yield ($Y_{x/i}$) values. We have revised the manuscript to explicitly address this issue in both the Results and Discussion sections.

In the Results, we now clarify that the biomass-yield coefficients are expressed in gDW per mmol and arise from the formulation of the unstructured multi-substrate model, serving as effective fitted parameters rather than classical physiological yield coefficients. In the Discussion, we further explain that these values reflect the distribution of biomass formation among simultaneously consumed substrates in a system where glycerol is the primary carbon and energy source, while most amino acids contribute mainly as nitrogen sources, precursors, or regulatory metabolites. As a result, relatively small amino acid uptake fluxes can be associated with substantial biomass accumulation driven by other substrates, leading to high apparent $Y_{x/i}$ values.

We also clarify that these coefficients do not impose unrealistic physiological behavior on the model and do not adversely influence its dynamic performance. Importantly, their relative ranking is consistent with experimentally observed consumption patterns and known metabolic roles of glutamate- and aspartate-family amino acids, supporting their usefulness as quantitative descriptors within the modeling framework.

Reviewer comment:

8. Overinterpretation of Regulatory Mechanisms (Lines 526–535)

The Discussion refers to nitrogen derepression, GlnR regulation, GS/GDH behavior, and urea-cycle contributions. Since no regulatory measurements were conducted, these points should be framed as literature-based context rather than conclusions drawn from this study.

Reviewer response:

We thank the reviewer for pointing out the potential overinterpretation of regulatory mechanisms. We have revised Lines 526–542 to clearly frame all references to nitrogen derepression, nitrogen catabolite repression, and the roles of GS, GDH, and related enzymes as established knowledge from the literature rather than as conclusions derived from the present study.

The revised text now explicitly attributes these regulatory mechanisms to previous studies and uses the experimentally observed amino acid consumption patterns solely to illustrate metabolic trends that are consistent with known nitrogen-dependent transitions in *Streptomyces*. Causal or mechanistic language implying direct regulatory inference has been removed, ensuring a clear distinction between experimental observations and literature-based physiological context.

Reviewer comment:

Overall Assessment

The manuscript is scientifically rigorous and effectively integrates experimental and computational approaches. It will be significantly strengthened with a clearer distinction between data and interpretation, a better explanation of the modeling components, and an acknowledgment of unexpected results and limitations of parameters.

I appreciate the depth of work presented and hope these comments contribute to enhancing both clarity and impact.

Reviewer response:

We sincerely thank the reviewer for the positive overall assessment of the manuscript and for recognizing the scientific rigor and integration of experimental and computational approaches. We have carefully addressed all comments by

clearly separating experimental observations from interpretation, improving the explanation of the modeling components, and explicitly acknowledging unexpected results and parameter limitations. We believe that these revisions have substantially improved the clarity, transparency, and overall impact of the manuscript, and we appreciate the reviewer's constructive feedback.

Response to Reviewer #2

We thank the reviewer for the positive assessment of the manuscript and for the constructive suggestions. All comments have been carefully addressed, as detailed below.

Comment 1

Figure captions should include more details (n value and statistical analysis if applicable).

Response:

Figure captions were revised to explicitly indicate the number of biological replicates (n = 3) for the experimental figures where applicable (Figures 2, 3, and 4). In addition, the Materials and Methods section was updated to clearly state that experimental measurements were obtained from three independent biological replicates. No inferential statistical analysis was applied, as the experimental data were used to parameterize and validate the dynamic metabolic model rather than for hypothesis testing.

Comment 2

Figure three is missing the x axis legend (Time in h).

Response:

The x-axis label in Figure 3 has been corrected to explicitly indicate time (hours).

Comment 3

If the resolution on figure 5 can be improved, it would increase legibility.

Response:

Figure 5 was regenerated at higher resolution to improve readability of network features and labels. The updated figure has been included in the revised manuscript.

Comment 4

Remove the image of a mitochondrion in Figure 7, which are not present in bacteria and thus misleading.

Response:

The mitochondrion image was removed from Figure 7. The figure now exclusively represents bacterial metabolic processes, ensuring biological accuracy and avoiding potential misinterpretation.

We believe these revisions have strengthened the clarity and accuracy of the manuscript and have fully addressed the reviewer's comments.
