# Supplementary material for: Dynamic metabolic modeling of Streptomyces clavuligerus in complex medium highlights nutrient-dependent metabolic transitions associated with clavulanic acid biosynthesis
Source: PLoS One. 2026 Feb 2;21(2):e0342057. doi: 10.1371/journal.pone.0342057 (PMC12863558; doi:10.1371/journal.pone.0342057)
Supplement: Attachment — Submitted filename: Reviewer comments- PONE-D-25-57065.docx [file pone.0342057.s004.docx]

The manuscript presents an integrative analysis of *Streptomyces clavuligerus* metabolism using experimental fermentation data, kinetic modelling, robustness analysis, network topology, and dynamic flux balance analysis. The work is technically rich, and the Discussion is particularly comprehensive, connecting amino acid utilization, nitrogen hierarchy, precursor fluxes, and metabolic switching. This systems-level perspective is valuable and less commonly seen in clavulanic acid studies. The English is clear and well written. I did not find any language issues that impede understanding

However, several areas of the manuscript would benefit from clarification and refinement to improve transparency, reproducibility, and scientific rigour. My comments are intended to be constructive, especially as a reader who is still learning these modelling approaches.

**Method Section-**

**1. Kinetic Modeling Description (Lines 137–144)**

The description of the unstructured kinetic model is too brief for readers unfamiliar with such models. Please include a short explanation of:

- how many differential equations were used,
- what each equation represents (biomass, glycerol, amino acids, CA, death),
- and the general parameter-fitting approach used.

Relying solely on S1 File makes the Methods section difficult to follow.

**2. Topological Analysis Workflow (Lines 146–161)**

The workflow for constructing the amino-acid network from the iLT1021 GEM is not sufficiently explained. Please clarify:

- how nodes and edges were defined,
- whether cofactors were excluded,
- how sequential reactions were collapsed into interactions.

This added detail would help non-expert readers understand how the network was built.

**3. Robustness Analysis and CA Production (Lines 163–178)**

It is unclear how CA production rates were obtained during Robustness Analysis. Since maximizing growth typically gives zero CA flux, I was unsure whether:

- a second optimization step was applied,
- CA fluxes were constrained, or
- the values were directly taken from the growth simulation.

**4. High Biomass-Yield Coefficients (Table 1)**

Some biomass-yield coefficients (e.g., **YX/Glu = 32.7**, **YX/Ala = 14.4**, **YX/Cys = 15.9 gDW/mmol**) are much higher than typical microbial yields. It is unclear whether:

- the units are correct (gDW/mmol vs. gDW/mol),
- the values reflect empirical fitting rather than physiological yields,
- or scaling/normalization effects are involved.

A short explanation in the Results or Discussion would help readers interpret these values correctly.

**Discussion Section**

The Discussion is thorough and integrates multiple analytical approaches. However, a few areas require clarification.

**5. Mixing Experimental Findings with Interpretation (Lines 464–472, 473–476, 477–487, 489–497, 508–516, 517–525)**

In several places, measured experimental data (e.g., Fig. 4 depletion trends) are presented together with physiological interpretations or model predictions. For example, Lines 464–472 combine measured amino-acid depletion with inferred causes such as anabolic demand or peptide hydrolysis.

A similar mixing appears in discussions of:

- nutrient triggers for stationary phase (Lines 473–476),
- hub-metabolite roles from topology (Lines 477–487),
- RA nitrogen-source performance (Lines 489–497),
- dFBA substrate and flux trends (Lines 508–525).

**Suggestion:** Please clearly separate observational results from interpretation, and explicitly indicate when statements derive from experimental data versus model predictions or literature reasoning.

**6. Missing Discussion of Unexpected Findings (Lines 473–487; 489–497; 273–279)**

Some noteworthy discrepancies are not addressed, such as:

- transient increases in certain amino acids early in growth,
- Lys/His showing poor CA support experimentally despite moderate predicted fluxes,
- low model fit for phosphate (R² = 0.423).

A brief explanation or acknowledgement would help contextualize these deviations.

**7. Interpretation of High Yield Values (Lines 282–297)**

Although high Yx values are discussed, the manuscript does not address:

- why these values exceed typical physiological limits,
- whether they result from model-fitting constraints,
- or whether they influence model behavior.

Please add a concise clarification.

**8. Overinterpretation of Regulatory Mechanisms (Lines 526–535)**

The Discussion refers to nitrogen derepression, GlnR regulation, GS/GDH behavior, and urea-cycle contributions. Since no regulatory measurements were conducted, these points should be framed as literature-based context rather than conclusions drawn from this study.

**Overall Assessment**

The manuscript is scientifically rigorous and effectively integrates experimental and computational approaches. It will be significantly strengthened with a clearer distinction between data and interpretation, a better explanation of the modeling components, and an acknowledgment of unexpected results and limitations of parameters.

I appreciate the depth of work presented and hope these comments contribute to enhancing both clarity and impact.
